# Increased use of cadaver specimens as a method for improving medical student satisfaction with clinical ear anatomy teaching at St George's, University of London: a pilot study

**DOI:** 10.1017/S0022215122000949

**Published:** 2023-01

**Authors:** M O'Hagan, D Sunnucks

**Affiliations:** 1 St George's, University of London, London, UK; 2 Head of Anatomy Queen Mary, University of London, Malta

**Keywords:** Education, Medical, Otolaryngology, Dissection, Students, Medical, Anatomy

## Abstract

**Background:**

Delivering sufficient otology education for undergraduates is known to be difficult, with limited teaching time being a contributing factor. Increasing student access to dissections of the ear could serve to increase satisfaction with teaching at St George's, University of London, UK.

**Objective:**

To evaluate student satisfaction with clinical ear anatomy teaching and investigate whether it can be improved using dissected specimens.

**Method:**

Participants completed an online survey and knowledge examinations, both before and after attending a new tutorial, with answers from before and after the session being compared.

**Results:**

Pre-teaching satisfaction scores concerning teaching were low, at an average of 2.45 (out of 7), with a mean examination result of 6.53 (out of 10). Post-teaching average satisfaction increased by 3.20 points to 5.65 (out of 7) (*p* < 0.01) and examination scores increased by 1.53 points to 8.07 (out of 10) (*p* < 0.01).

**Conclusion:**

Students are supportive of increased access to cadaver dissections of the ear, and facilitating this can improve satisfaction with otology teaching.

## Introduction

ENT is an important specialty; it forms a significant part of the surgical workload, being the fourth largest surgical specialty in the National Health Service.^1^ Despite this, exposure to ENT surgery at the undergraduate level has been found to be minimal,^2^ with 58 per cent of ENT attachments during medical school being combined with other specialties, including dermatology, ophthalmology and neurology.^3^

The majority of final year medical students and junior doctors do not feel they have been adequately trained to assess and manage ENT patients.^4–6^ A lack of consistency across UK medical schools with regard to their undergraduate ENT curriculum further compounds these shortcomings,^7^ which is concerning as this stage of training is crucial in encouraging students to pursue a career in ENT.^8^ Furthermore, it was found that 30 per cent of general practitioners in South West England had received no formal hospital experience or had any form of post-graduate teaching in ENT.^9^

In conjunction with this, anatomy should form an integral part of any surgical education, yet only 53.8 per cent of newly qualified doctors felt they had received adequate anatomy teaching in medical school.^10^ Medical students have also been considered, and approximately 50 per cent of students rated their own anatomical knowledge as inadequate to be a competent foundation year one doctor.^11^ This represents a UK-wide shortfall in ENT teaching at both undergraduate and post-graduate levels.

Consideration is therefore needed regarding how ENT undergraduate education can be improved in a system that is becoming increasingly time-scarce. The principles of cognitive flexibility theory could be applied, whereby ear anatomy would be taught through multiple different modalities such as plastic models, dissected specimens and radiographical images of the ear.^12,13^ Ferguson *et al*. recommend a blended approach to ENT teaching involving both online resources and clinical placements.^2^ This has been effective in increasing student satisfaction within other allied healthcare subjects,^14^ and otolaryngology trainees specifically have demonstrated receptiveness to additional e-learning modules as an adjunct to their training.^15^

Traditionally, anatomy teaching at medical schools has relied on cadaveric dissection as a method to give students a hands-on view of the body.^16^ Whilst St George's, University of London, UK, uses cadaver dissections and specimens to teach gross anatomy, there are currently very limited dissections of the ear available. With a progressive decrease in teaching hours, learning is moving away from traditional, dissection-based classes towards plastic models and computer-based teaching.^17^ This has been identified as having a negative influence on students’ anatomical knowledge, as learning anatomy through active exploration contributes to improved learning and subsequent anatomical knowledge.^18^

Prosections act as a compromise for medical schools, in that they can still deliver anatomy teaching using cadavers and thus benefit from the human form, whilst also reducing time spent training students in the art of dissection. However, research into whether dissection can be replaced by alternative modalities such as computer-aided learning, three-dimensional models or tutorials continues to draw a common conclusion: medical students like dissection.^19–22^

The primary objective of this study was to evaluate whether a new teaching session incorporating greater access to cadaver specimens of the ear can improve participant satisfaction with otology teaching and examination performance. The secondary objective of this study was to evaluate wider student opinion on current ear anatomy teaching at St George's, University of London.

## Materials and methods

### Participant eligibility

Those medical students at St George's, University of London in their first, second or third clinical year were eligible to enrol in the study. Participants were recruited via an e-mail advert sent to all eligible students, and via a targeted social media campaign. All participation was voluntary, and participants were offered access to additional study material for completion of the study.

An arbitrary difference of 1.5 points (out of 7) or more in the mean Likert-type scale responses between the pre-teaching and post-teaching survey was selected to represent a meaningful effect. Power calculations suggested that a sample size of 18 students completing each survey would be necessary to detect this difference in means at a significance level of 95 per cent and with a power of 0.95.

This study was given a favourable ethical opinion by the Joint Research and Enterprise Services at St George's, University of London (research ethics committee reference: 2020:0286).

### Study design

Participants were first allocated to one of two study streams depending on their time availability. Primary stream participants would complete all aspects of the study, with the aim of addressing the primary objective. Secondary stream participants would complete a single online survey, with the aim of addressing the secondary objective. Splitting participants into two streams would give students who did not have enough time to complete the full study the opportunity to partly contribute, and therefore provide a larger sample size.

Student opinion regarding teaching was first evaluated in both study streams through an online survey, which covered topics such as volume of previous ear anatomy teaching, confidence in their own knowledge and satisfaction with previous teaching.

Next, primary stream participants’ current knowledge was objectively assessed through an anatomy spotter examination, which tested both ear anatomy and its clinical associations. The anatomy spotter examination was hosted on a virtual platform and contained five stations, with each station containing two questions. The first question of each station asked participants to identify an anatomical structure related to the ear on an image displayed on their screen, with a follow-up question about clinical relevance. This image was of either a dissected specimen, a diagram or a plastic model. This allowed both anatomy and clinical knowledge to be assessed simultaneously.

Following completion of the first survey and the examination, participants were invited to watch two lectures covering ear anatomy and associated clinical aspects. In light of changing coronavirus disease 2019 restrictions at the time of this study, five participants attended the new teaching session onsite, with the remainder attending an online version of the session.

These lectures provided greater access to ear dissections, with the online version utilising microscope images of a new dissection, produced with the help of St George's ENT surgeons and the St George's, University of London anatomy department. The onsite session occurred before the new dissection was performed, and therefore participants had physical access to two pre-existing dissections that were available in the anatomy department. The session was based on learning objectives^23^ that were broadly akin to the following: (1) ‘describe the intracranial and intrapetrous course of the facial nerve and the relationships of its major branches to the middle ear in relation to damage of the nerve within the facial canal’; and (2) ‘describe the functional anatomy of the auricle, external auditory meatus, tympanic membrane, auditory ossicles and pharyngotympanic tube’.

Finally, students re-took the survey and anatomy spotter examination so that their knowledge and satisfaction from before and after the session could be compared. These quantitative data were statistically analysed in IBM SPSS® software, version 28.0.

Both the pre- and post-teaching survey contained nine discrete Likert-type questions that were combined to form a Likert-type scale. This scale generated a ‘satisfaction’ score for each participant, and these pre- and post-teaching scores were compared. Other survey items were included in the survey that were not utilised as part of the Likert-type scale. Details of reported questions included in the survey can be seen in the results section of this paper.

In order to evaluate the primary objective of the study, two endpoints were identified. The primary endpoint of the study was the difference in means between the participants’ pre-teaching satisfaction and post-teaching satisfaction scores. The secondary endpoint of the study was the proportion of participants who believed increased exposure to dissected specimens would aid their learning.

### Statistical analysis

Both Likert-type scales were analysed as continuous variables.^24,25^ The first quartiles, medians and third quartiles were calculated for each Likert-type question included in the scales. A ‘satisfaction’ score was calculated from each Likert-type scale by calculating the mean participant response for each question included, and then calculating an overall mean for the series of questions to produce a pre- and post- teaching score. Standard deviation (SD) and Cronbach's alpha were calculated for each scale.

A paired *t*-test was performed to compare pre- versus post-teaching examination results, and pre- versus post-teaching Likert-type scale responses.^26^ Relative SD was calculated for both sets of examination results. The Shapiro–Wilk test of normality was used to assess data normality.^27^

## Results

Twenty-seven participants were recruited to the primary stream of this study. Only participants who completed both the pre- and post-teaching survey or pre- and post-teaching examination were included in the primary stream analysis, with the rest being transferred to the secondary stream if applicable. Subsequently, 19 participants completed the pre- and post-teaching survey, and 15 completed the pre- and post-teaching examination. An additional 19 participants were recruited anonymously to the secondary stream, as well as 23 who completed the pre-teaching survey as part of the primary stream. Therefore, 42 participants completed the pre-teaching survey to investigate the secondary objective.

### Likert-type scale analysis

**Fig. 1.**
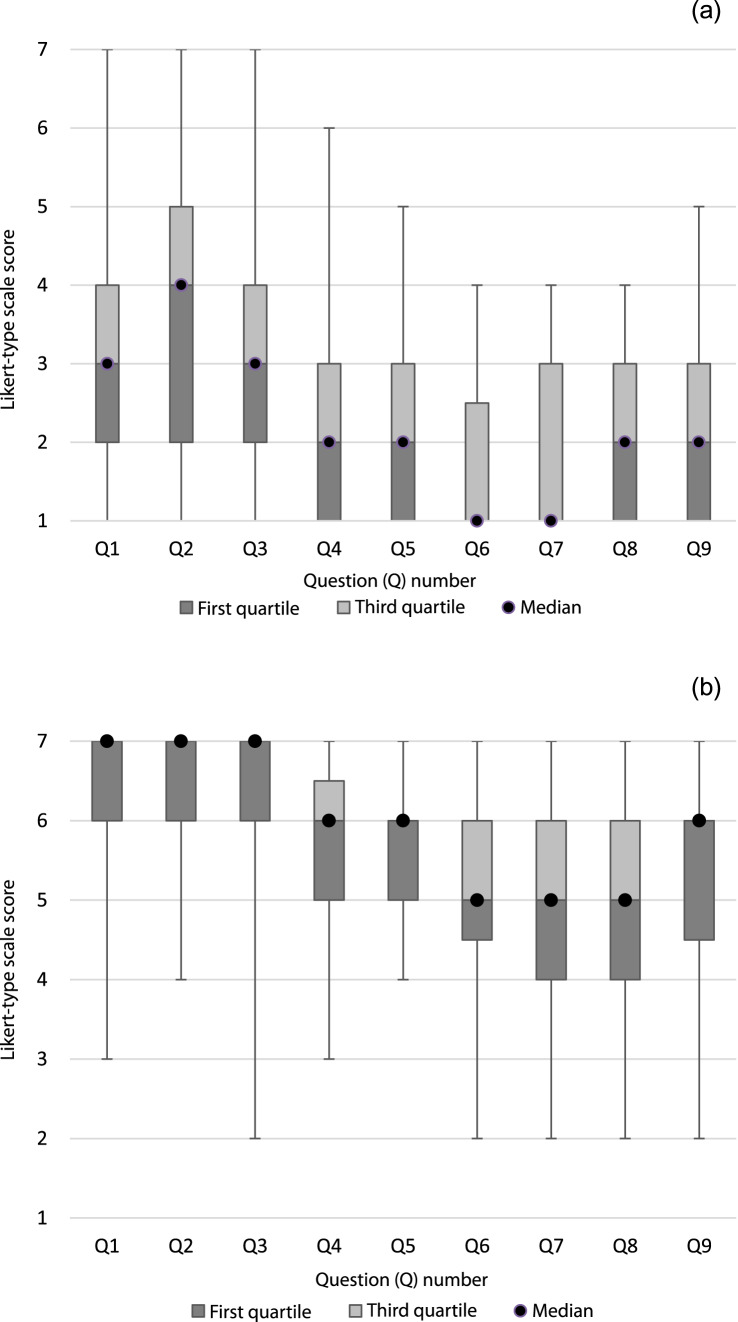
Box plot chart displaying participants’ responses to questions included in (a) the pre-teaching and (b) the post-teaching Likert-type scales. Question 1 = ‘How successful were previous teaching sessions at meeting your learning objectives on clinical ear anatomy?’; question 2 = ‘How satisfied were you with the quality of previous teaching by tutors on clinical ear anatomy?’; question 3 = ‘How useful were the previous teaching sessions to your studies on clinical ear anatomy?’; question 4 = ‘How confident are you that you know what is required of you regarding your ear anatomy learning objectives?’; question 5 = ‘How confident do you feel in your understanding of clinical ear anatomy?’; question 6 = ‘How confident do you feel currently in explaining ear anatomy to a colleague?’; question 7 = ‘How confident do you feel currently in explaining ear anatomy to a patient?’; question 8 = ‘How confident do you feel currently in explaining ear pathology to a colleague?’; and question 9 = ‘How confident do you feel currently in explaining ear pathology to a patient?’.

Cronbach's alpha was 0.838 and 0.905 for the pre-teaching and post-teaching Likert-type scale score respectively. Normality testing produced a pre-teaching significance of 0.422 and post-teaching significance of 0.056. Although the post-teaching survey results were approaching significance (< 0.05), they were analysed using parametric tests because of the nature of the scale and the small sample size.^25^ The median, first quartile, third quartile and range for participant responses to questions included in the Likert-type scales are shown in Figure 1.

Paired *t*-testing revealed a statistically significant increase in mean satisfaction with teaching in the post-teaching (mean = 5.65, SD = 0.945) compared with the pre-teaching Likert-type scale score (mean = 2.45, SD = 0.900) of 3.20 points (95 per cent confidence interval (CI) = 2.54–3.86; *p* < 0.01).

### Examination results analysis

Shapiro–Wilk normality testing returned significance values for the pre-teaching and post-teaching examinations of 0.113 and 0.333 respectively, confirming that the data could be analysed parametrically.

**Fig. 2.**
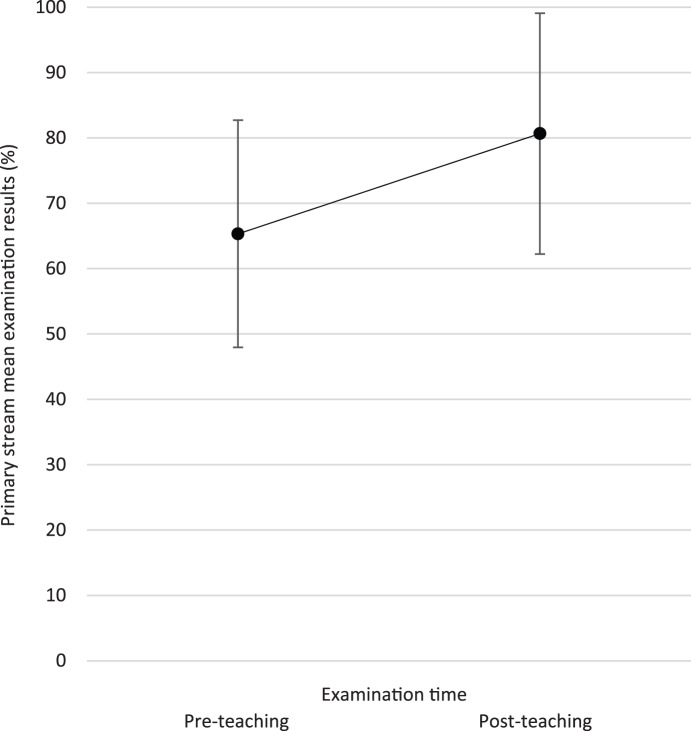
Line chart displaying the primary stream mean results (and error bars, representing relative standard deviation (SD)) from the pre-teaching (65.3 per cent (relative SD = 17.23 per cent)) and post-teaching (80.7 per cent (relative SD = 18.43 per cent)) examinations.

The average examination result improved from the pre-teaching examination (mean = 6.53 (out of 10), SD = 1.125) compared with the post-teaching examination (mean = 8.07 (out of 10), SD = 1.486) by 1.53 points (95 per cent CI = 0.45–2.62). Paired *t*-testing showed this to be statistically significant (*p* < 0.01). Figure 2 displays the mean examination scores from before and after teaching.

### Survey responses

The survey showed that 76.2 per cent of students had received 2 hours or less of formal teaching on clinical ear anatomy, with 76.2 per cent of students believing this formal teaching time was not sufficient to meet their learning objectives. Further to this, it was found that 64.3 per cent of students had spent less than 5 hours studying clinical ear anatomy independently. Participants suggested that, on average, 3.7 hours of formal teaching was required to meet their learning objectives, which would equate to at least one further hour-long teaching session on top of previous teaching.

Opinion on how that additional teaching should be delivered is divided. Participants were able to indicate their preference for different teaching methods from a list of options, with free text also available. Their responses included: a clinical skills small-group teaching session (27.2 per cent); a dissection room session with either plastic models (27.2 per cent) or prosections (25.0 per cent); a lecture format (10.9 per cent); or an online learning module (9.8 per cent). Of the responses, 79.4 per cent related to some form of small-group teaching session.

## Discussion

**Fig. 3.**
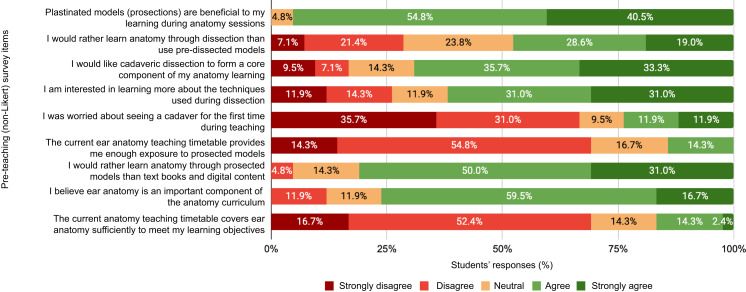
Bar chart showing students’ responses (*n =* 42) to survey items included in the pre-teaching survey that were not used to form the Likert-type scale.

Students recognise the importance of otology within their curriculum (Figure 3); however, their satisfaction with current teaching is low, as evidenced by the pre-teaching satisfaction score of 2.45 (out of 7). Only 16.7 per cent of students believe the current teaching timetable is sufficient to meet their learning objectives. This is consistent with similar literature, where it was reported that 71 per cent of participants felt teaching of head and neck anatomy at the undergraduate level was inadequate.^28^ Organising and producing a new lecture with greater exposure to dissected specimens led to an increase in students’ satisfaction score to 5.65 (out of 7).

Analysis of the individual questions included in the Likert-type scale provides further insight into how confident the students are in their clinical ear anatomy knowledge. Students were asked to rate their confidence in explaining ear anatomy to both a patient (median response of 1 (out of 7)) and a colleague (median response of 1 (out of 7)) in the pre-teaching survey, to gauge their understanding of these learning objectives, and to bridge the gap between the classroom and clinical environment (Figure 1). These very low scores suggest the knowledge they have is shallow and lacks deeper understanding. As 76.2 per cent of students report receiving only 2 hours or less of formal teaching, this would suggest that a lack of teaching time is a factor.

**Fig. 4.**
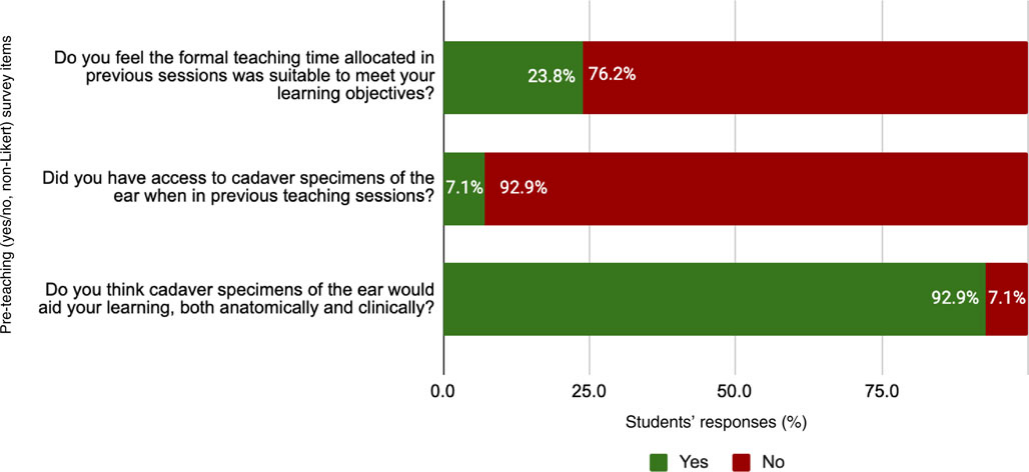
Bar chart showing students’ responses (*n =* 42) to yes/no survey items included in the pre-teaching survey that were not used to form the Likert-type scale.

Furthermore, the students’ responses show they favour cadaver-based learning with prosections over more generic approaches like lectures and online learning modules (Figure 3). Of students, 92.9 per cent agreed that cadaver specimens of the ear would be beneficial to both anatomical and clinical teaching (Figure 4). In contrast, only 14.3 per cent of students felt they had adequate access to prosections of the ear during their teaching (Figure 3). Providing greater access to such material during already established teaching sessions could therefore increase student satisfaction without the need to schedule additional sessions.

Participants not only supported the use of cadavers in learning anatomy, but responses showed a preference for prosected models (81.0 per cent) over digital content and textbooks. A further 95.3 per cent of students ‘agreed’ or ‘strongly agreed’ that prosections are beneficial to their anatomical learning (Figure 3). Cadaver use during anatomy teaching has been shown to increase long-term retention of knowledge versus more modern approaches,^29^ suggesting that the benefits of cadaver use early on in training may be sustained into students’ clinical practice.

Interestingly, 47.6 per cent of participants ‘agreed’ or ‘strongly agreed’ that they would rather learn anatomy through active dissection than utilise pre-dissected models. Only 23.8 per cent ‘agreed’ or ‘strongly agreed’ that they were worried about seeing a cadaver for the first time, and 62.0 per cent ‘agreed’ or ‘strongly agreed’ that they were interested in learning more about the techniques used during dissection (Figure 3). This suggests that students prefer to learn through exploration, rather than having ‘pre-planned routes’ for them to study from. They would rather learn methodically, dissecting through different layers and experiencing the human form for themselves, than have a diagram or textbook drawn for them by someone who has likely already had dissection experience.

One explanation for a preference towards cadaver-based learning could be that medical students are required to apply their anatomical knowledge on placements with patients. Cadavers therefore provide an almost like-for-like experience in learning anatomy, which can then be applied in the clinical context. It is recognised that intricate dissection of the ear requires a high level of skill, one that is likely out of reach for most undergraduate students. A solution to this could be guided tutorials where surgeons dissect through the ear and teach the anatomy as they go, which could be employed as an e-learning module complimentary to clinical placements. This has been trialled in undergraduate clinical education by ENT surgeons recently, where it was found to enhance students’ learning experience.^30^

Participants were also asked for their opinion on how the current teaching timetable could be improved. Of the 28 responses, 20 (71 per cent) were related to increasing the amount of time dedicated to the ear specifically. Select responses from students included:

‘We don't have specific ear anatomy teaching regarding the different parts of tympanic membrane, nerve, sound conduction, etc. It would be beneficial to dedicate a session just for ear anatomy as it is not covered in Head and Neck and it often comes up in both examinations and clinical practice’.

‘Need more time, we had two sessions to study the entirety of the head and neck, the ear got lost somewhere in there’.

‘More time going through the clinical skills of an ear, nose and throat examination as well as being able to recognise and present back common ear pathology’.

A doctor requires not only clinical knowledge, but also strong communication skills to discuss this information with patients in a digestible manner. Providing participants with an extra 1.5 hours of teaching with increased cadaver specimen exposure, focused on essential anatomy and the associated pathology, increased students’ confidence in explaining ear anatomy to both a patient and a colleague (Figure 1). This indicates the benefit of introducing more dissections and more formal teaching into the timetable. Further to this, confidence in explaining ear pathology to both patients and colleagues was improved, reinforcing the importance of anatomy as a foundation to both pre-clinical and clinical education.

One possible intervention for improving satisfaction further is to introduce the teaching as a communication and clinical skills session. This could incorporate elements of explaining pathology to a patient and answering any questions they may have. A significant improvement in objective structured clinical examination performance can be seen in students who receive such communication training.^31^

### Developments for future

The integration of new and innovative teaching methods has been shown to improve not just satisfaction with teaching, but also examination performance. Such innovations include computerised three-dimensional (3D) models,^32^ holographic models,^33^ holographic lenses^34^ and even virtual reality tutors.^35^ Nicholson *et al*., used a high-resolution magnetic resonance imaging scan of the middle and inner ear to construct a 3D model that was then used for teaching.^36^ The intervention group of students had the new 3D model integrated into their learning, and a control group did not. The intervention group scored significantly higher on subsequent examinations, demonstrating the possible value in these new teaching tools.^36^

However, it remains that cadaver dissections are an appreciated and effective learning method for students. This favouring by students for cadaver-based learning is at odds with medical schools’ decisions to lean away from dissection because of economic pressures and time constraints. A combined approach utilising cadavers and prosections alongside digital content would form a suitable compromise requiring less formal timetable space, whilst still satisfying students and providing them the opportunity to learn from different mediums. This is not a new idea and has been discussed in the literature previously.^37^ This is further reinforced by Memon, who undertook a strong literature review summarising these points.^38^

A good starting point for developing the ear anatomy curriculum further would be to make cadaver specimens of the ear more readily available to students. This could be integrated into an existing anatomy session in the dissection room or be part of a new teaching session. A combined approach of increased teaching time alongside greater access to cadaver specimens could therefore represent an improvement to current teaching at St George's, University of London.

### Limitations and improvements

The sample size of this study is small. A larger scale study investigating a whole medical cohort will provide more reliable results, and will encompass a larger sample size of views and opinions. This could be carried out as a randomised, controlled trial, with half of a student population receiving teaching involving cadaver specimens, and the other not. Satisfaction could then be compared using surveys to establish how successful each cohort's teaching was.

The online lecture format presented some problems that may have affected students’ satisfaction with the sessions. A lack of direct engagement with students made it difficult to ensure that material was being covered correctly and adequately. Online classes inherently increase personal responsibilities on students, making it harder for teachers to ensure that learning material is being covered fully.^39^ An even greater improvement may have been seen if the session was conducted in small groups, if the session was conducted in small groups, with direct cadaver access.

Detailed feedback can help improve engagement, and is important in guiding students’ development, and in providing motivation to deepen their understanding of key topics through associated feelings of reward versus punishment.^40^ Feedback was not given as part of the study, in either the teaching session or examination; this could be implemented to improve student engagement. Social media can also be utilised to improve engagement and promote camaraderie between students, to create a sense of community.^41^ The social aspect of learning is important to students' educational development, as learning is mostly a socio-cognitive activity.^42^

The website data indicated that students spent on average only 4 minutes completing the examination. This suggests that either the questions were too straightforward, or that students were not fully engaged in the material and rushed through it. Further research could therefore utilise a real examination environment and incorporate formative ear anatomy questions as part of a larger summative examination. This way, students would have greater motivation to engage with the questions.

- ENT remains an under-represented specialty in the undergraduate curriculum given its clinical significance within the National Health Service
- Medical student satisfaction with otology teaching at St George's, University of London is currently low
- Improving access to cadaver specimens of the ear can increase satisfaction with teaching and confidence in fundamental ENT knowledge
- Medical students value cadaver use in ENT teaching, providing a foundation to build clinical knowledge
- Cadaver-based sessions can be delivered through a variety of modalities, allowing flexibility and adaptability when tailoring teaching around a busy timetable

The move to online teaching made the provision of in-person access to cadaver specimens difficult. This resulted in less cadaver interaction than was originally planned. Future research could deeper engrain cadaver specimens into the session and provide more opportunities for students to examine these specimens for themselves.

## Conclusion

Student satisfaction with ear anatomy teaching is currently low. This could potentially be improved by increasing the amount of time allocated to formal teaching. This study demonstrates that increasing the availability of cadaver specimens of the ear for students to learn from could significantly increase satisfaction with teaching. New innovations for teaching anatomy such as virtual dissection tutorials and holographic lenses provide exciting avenues, whereby greater cadaver access can be incorporated into the ENT curriculum. Further research is needed regarding their efficacy as teaching tools and to assess students’ perceptions of learning in this way.
